# UNAIDS 90-90-90 Target and Retention in Care of a Cohort of Migrants Living with HIV in a Tertiary Referral Hospital in Florence, Italy

**DOI:** 10.1007/s10461-025-04716-9

**Published:** 2025-05-06

**Authors:** Giuseppe Gasparro, Sasha Trevisan, Seble Tekle Kiros, Costanza Malcontenti, Michele Trotta, Anna Barbiero, Beatrice Borchi, Filippo Bartalesi, Paola Corsi, Costanza Fiorelli, Gaetana Sterrantino, Alessandro Bartoloni, Filippo Lagi

**Affiliations:** 1 Department of Experimental and Clinical Medicine, University of Florence, Florence, Italy; 2 Infectious and Tropical Diseases Unit, Careggi University Hospital, Florence, Italy; 3 Infectious Diseases Unit, Santa Maria Annunziata Hospital, AUSL Toscana Centro, Bagno a Ripoli, Italy

**Keywords:** HIV, Italy, Migrant, UNAIDS goals, Continuum of care, Loss to follow up

## Abstract

Migrant Living with HIV (MLWH) are facing many barriers. Proposing targeted interventions requires a better understanding of the local epidemiology, but data are scarce. This population often comprises vulnerable groups such as men who have Sex with men and transgender individuals. This single-center cohort study aims to estimate the achievement of the Joint United Nations Programme on HIV/AIDS 90-90-90 goals and the 8-year loss-to-follow-up (LTFU) incidence rate in a cohort of MLWH under treatment at the Infectious and Tropical Diseases Unit of the “Careggi University Hospital”, Florence, Italy. We enrolled MLWH taken in care from 01/01/2014 to 31/12/2022. The end of the study was the end of follow-up (30/04/2023) or the date of LTFU (unreachable, relocated to another center, or dead). We enrolled 201 migrants with a median age of 33 [IQR 27–43]. One-hundred-and-six (52.7%) came from Latin America, mainly from Peru (40.2%; *n* = 81). About a third were transgender women (TW) (32.8%; *n* = 66). Seventy-six (37.8%) were migrants out-of-status (MOS). HIV was diagnosed in Italy in 58.7% (*n* = 118). Ninety (44.8%) were treatment-naïve, sex-working was reported in 39 patients (19.4%) before and 55 (27.4%) after migration. One-hundred-thirty-eight (68.7%) were retained in care. The 8-year-LTFU incidence rate was 8.96 per 100 p/y (95% CI 7.0–11.4). MOS had a higher risk of LTFU (aHR 2.68; *p* = 0.005). Conversely, being a TW (aHR 0.33; *p* = 0.024) and taking a single-tablet-regimen (aHR 0.44; *p* = 0.008) were protective factors.In our setting the 90-90-90 targets have not yet been fully achieved, and high rates of LTFU have been observed.

## Background

Population movements are a complex phenomenon that involves several aspects including health. According to the World Migration Report there are 281 million international migrants globally, the 3.6% of the world’s population, and Europe is currently the preferred destination for international migrants, with 87 million migrants (30.9%), followed closely by migrants to Asia (30.5%) [1–2].

Migration drivers are numerous, including poor living conditions, violence, environmental issues, fundamental rights violations, and the widening gap between high- and low-income countries [3]. Migrants often comprise vulnerable groups such as MSM (Men who have Sex with Men) and transgender individuals who often migrate in search of less discriminatory environments but continue to face significant challenges post-migration, including job, health, and social discrimination [4]. Post-arrival, migrants contend with cultural and language barriers, familial separation, and hurdles in employment and healthcare, which often lead to marginalization and sometimes force them into illegal work or prostitution to finance their journey. This vulnerability places them at higher risk of social, political, and economic marginalization, as well as infections like HIV [5–6].

The European Centre for Disease Prevention and Control (ECDC) reports that migrants are disproportionately affected by HIV, representing 42% of new diagnoses in the EU in 2021 and 48% in 2022 [7]. Diagnosis is often in the advanced stages of disease and migrants are more likely to be lost to follow-up [8]. Migrants living with HIV (MLWH) experience compounded stigma associated with their disease, migration status, and often racial and cultural discrimination, which is even higher for MSM post-migration [9].

The Joint United Nations Programme on HIV/AIDS (UNAIDS) set forth the 90-90-90 goals within the HIV Care Continuum framework: 90% of People Living With HIV (PLWH) should know their status, 90% of those diagnosed should receive antiretroviral-therapy (ART), and 90% of those on treatment should achieve viral suppression [10–11]. Furthermore, UNAIDS has introduced the ambitious 95-95-95 targets, aiming to end HIV/AIDS as a public health threat by 2030 [12]. Attaining these targets for MLWH, especially in terms of viral suppression, is challenging. In Italy, data disaggregation for this group is scarce [13], but it is clear that migrants have a higher HIV prevalence than native populations necessitating tailored and inclusive health strategies [6]. High-mobility individuals are particularly prone to higher rates of virological failure and loss to follow-up [14–15–16], complications that the pandemic has exacerbated [17–18]. Data on these groups are scant, partly due to their underrepresentation in clinical studies and reluctance to engage in research, creating barriers to understanding and addressing their needs [19–20]. This analysis aims to deepen our understanding of HIV care continuum dynamics among migrants attending a large HIV clinic in central Italy to design targeted interventions, facilitate better access to healthcare, ameliorate retention in care, and improve virological success rates.

## Study Design, Setting and Aim

We conducted a monocentric retro-prospective observational cohort study. We collected anonymized data from outpatient records of all adult MLWH, with their first outpatient visit between 01/01/2014 and 31/12/2022, taken in care at the Infectious and Tropical Diseases Unit of the “Careggi University Hospital”, a referral tertiary care center in Florence, Italy, treating about 1600 PLWH.

Italy, a major European entry point for migrants, had over 3.5 million legally residing non-EU citizens as of January 2022. Florence stands out with a non-EU resident percentage of 10.1%, of which 6.8% are from Peru, surpassing the Italian average. Moreover, in 2022, Florence saw an 11.7% increase in non-EU presence, higher than the national average [21].

The primary aim of our study was to describe the migrant population taken in care in our clinic and to estimate the continuum of care in migrants according to the 90-90-90 UNAIDS goals. Secondary outcomes were to estimate the incidence rate of loss to follow-up at 8 years and to determine factors predictive of loss to follow-up.

### Definitions and Inclusion Criteria

We enrolled all MLWH aged > 18 years, therapy naïve and experienced, with at least two medical visits at the Infectious and Tropical Diseases Unit of the “Careggi University Hospital” between 01/01/2014 and 31/12/2022. We defined a MLWH as a foreign-born individual with a confirmed diagnosis of HIV infection who has been living in Italy for at least six months. Extra-European migrants who entered Italy without control or arrived regularly but whose entry or residence permit has expired can request the STP (Temporarily Present Foreigner) code. The STP code is issued following a declaration of indigence and guarantees urgent health services free of charge. It is valid throughout the Italian national territory, lasts six months and is renewable. We have defined all those MLWHs in the latter condition as “out of status”. The enrolment was not dependent on formal documentation and immigration status. An HIV viral load < 50 copies/mL was defined as undetectable. We tracked patients until their last recorded visit to the center. Those not seen for a year after their last medical visit due to loss of contact, transfer to other healthcare facilities, or death were considered lost to follow-up (LTFU). All patients classified as “unreachable” were defined as such only after having made one phone call per day for three consecutive days without receiving any response to the phone numbers provided by the participants at the time of enrolment. The data cut-off was April 30, 2023. All information was gathered via the center electronic patient health records.

### Ethics

The study was approved by the local ethics committee (Protocol number 16283_oss), in accordance with the International Conference on Harmonization Good Clinical Practice (ICH GCP) and the Declaration of Helsinki, respecting ethical principles and good clinical practice. All enrolled patients provided a written informed consensus for recording their data for research aims.

### Statistical Analysis

We used descriptive statistics such as medians, with their corresponding interquartile ranges (IQR), to describe continuous variables and proportions for categorical variables. Parametric (chi-square, Fisher’s exact test) and non-parametric (Mann-Whitney) tests were used where indicated. For multivariate analysis, the Cox’s regression model was used to identify factors associated with loss to follow-up. The entry point into the cohort was considered the first visit to the medical center. The end of the observation period was considered the last visit at the center, or the date of loss to follow-up or the patient’s death. The cumulative risk of loss to follow-up was assessed using Kaplan–Meier curves. Associations with a p-value < 0.05 were considered statistically significant. Data was analyzed using Stata’s statistical program (Version 14.0, StataCorp, College Station, Texas).

## Results

**Fig. 1:**
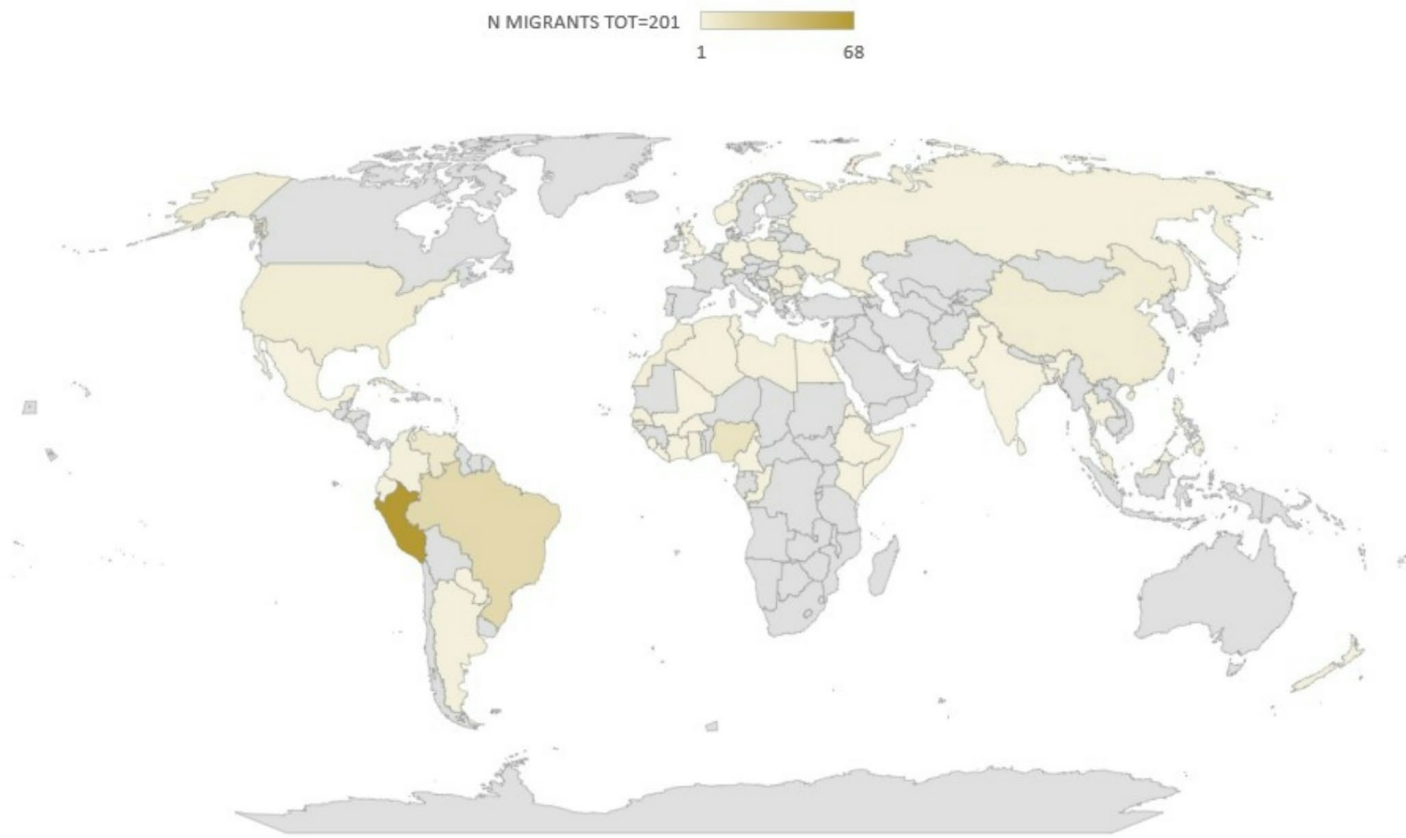
Country of origin of a migrant population living with HIV taken in care from 01/01/2014 to 31/12/2022 (*N* = 201) at the Infectious and Tropical diseases unit in a tertiary teaching hospital in Florence, Italy

Our study enrolled 201 migrants, with a median age of 33 years [IQR 27–43 years]. Among them, a significant proportion (*n* = 66; 32.8%) were transgender women. The predominant risk factor reported was unprotected sex among MSM, which accounted for more than half of the cases (*n* = 112; 55.7%). A notable 37.8% (*n* = 76) of the migrants were out-of-status, while 19.4% (*n* = 39) had engaged in sex work before migration and 27.4% (*n* = 55) after migrating. Of these, 36 reported engaging in sex work both before and after migration. Over half of the cohort originated from Latin America, mainly from Peru (Fig. 1).

In our cohort, more than half of the HIV infections (*n* = 118; 58.7%) were diagnosed in Italy, while the remaining 41.3% (*n* = 83) received their diagnosis abroad. Among those diagnosed outside Italy, 77 were ART-experienced, with 7 of them not receiving ART at the time of their enrolment.

On their first visit, 44.8% (*n* = 90) were naïve. Among the experienced patients (*n* = 111; 55.2%), 44.1% (49 out of 111) were not virologically suppressed at their initial clinic visit. By the study’s conclusion, about 75% (*n* = 151) were on a single tablet regimen (STR). Of those lost to follow-up (*n* = 63), 53.9% (*n* = 34) were unreachable, 38.1% (*n* = 24) had transferred to another treatment center, and one was dead. All transferred patients were referred to other health centers in Italy.

**Fig. 2:**
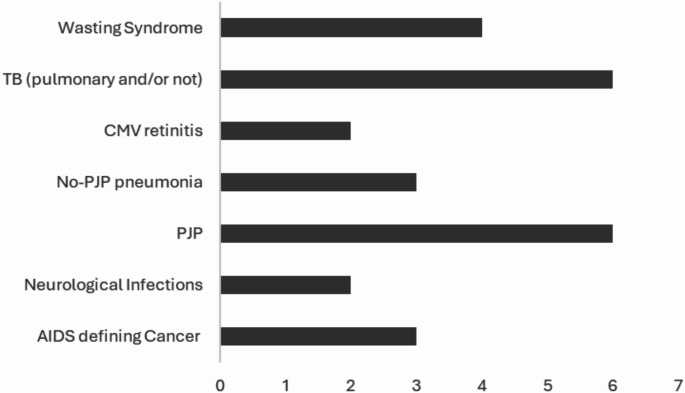
Number of AIDS defining diseases cases recorded in the medical history in a cohort of migrant People Living With HIV taken in care from 01/01/2014 to 31/12/2022 (*N* = 201) at the Infectious and Tropical diseases unit in a tertiary teaching hospital in Florence, Italy. *TB* tuberculosis, *CMV* cytomegalovirus, *PJP P. jirovecii* Pneumonia, *AIDS* acquired Immunodeficiency Syndrome, Neurological infections include neurotoxoplasmosis and cryptococcal meningitis; AIDS-related cancers include Kaposi’s sarcoma and Burkitt’s lymphoma

We found 19.4% (*n* = 39) of the patients enrolled with a medical history of Acquired Immunodeficiency Syndrome (AIDS) (Fig. 2). Complete descriptive analysis of clinical, laboratory and demographic characteristics is shown in Table 1.

**Table 1:** Clinical, laboratory, and demographic characteristics of a migrant population living with HIV taken in care from 01/01/2014 to 31/12/2022 (*N* = 201) at the infectious and tropical diseases unit in a tertiary teaching hospital in Florence, Italy

	N (*n* = 201)	% [IQR]
Gender (N, %)		
Cisgender Men	91	45.3
Cisgender women	44	21.9
Transgender women	66	32.8
Naïve patients (N, %)	90	44.8
Age in years at first visit (median, IQR)	33	27–43
Mode of transmission (N, %)		
Unprotected sex (heterosexual)	72	35.8
Unprotected sex (MSM)	112	55.7
Vertical transmission	4	2.0
IVDU and MSM	3	1.5
Transfusion	2	1.0
Unknown	8	4.0
Migrants incoming geographic area (N, %)		
Latin America	106	52.7
Africa (excluded Sub-Saharan)	31	15.4
East-Europe	28	13.9
Asia	16	8.0
Sub-Saharan Africa	10	5.0
West Europe	5	2.5
North America	4	2.0
Oceania	1	0.5
Years between arrival in Italy and linkage to care (median, IQR)	2	0.3–11
Migrants out-of-status on first visit (N, %)	76	37.8
First HIV positive result performed in Italy (N, %)	118	58.7
Smoke (N, %)	54	27
Sex working (N, %)		
Pre-migration		
Yes	39	19.4
No	89	44.3
Unknown	73	36.3
Post-migration		
Yes	55	27.4
No	79	39.3
Unknown	67	33.3
IVDU pre/postmigration (N, %)		
Yes	4	1.9
No	149	74.1
Unknown	48	24.0
AIDS diagnosis (N, %)	39	19.4
Acute infection (N, %)	22	10.9
Nadir CD4 cells/mmc (median, IQR)	393	165–582
Zenith viral load, Log _10_ (median, IQR)	4.7	3.3–5.3
CD4 at baseline cells/mmc (median, IQR)	490	235–750
Viraemia of naïve patients at first visit, Log _10_ (median, IQR)	4.89	4.37–5.36
Days between linkage to care and art initiation in naïve patients (median, IQR)	12	4–39
Patients experienced with viraemia > 50 cp/mL at first visit (N, %)	49 out of 111	44.1
STR at the end of the study (N, %)	151	75.1
Loss to follow up causes (N, %)		
Transfer to another treatment center	24	38.1
Unreachable patient	34	53.9
Death	1	1.6
Other^b^	4	6.4
Resistance mutation on genotype at baseline (N, %)^a^		
None	95	47.2
Not performed	73	36.2
Yes	33	16.4
RT (reverse transcriptase)	30	23.4
PR (protease inhibitor)	5	3.9
INSI (integrase strand inhibitor)	1	0.7

*IQR* interquartile range, *MSM* males who have sex with males; *IVDU* intravenous-drug users; *OUT-OF-STATUS* including arrived regularly but whose VISA has expired, *HBsAg* hepatitis B surface antigen; *HBcAb* hepatitis B core antibody; *HCV* hepatitis C virus; *HAV* hepatitis A virus, *AIDS* acquired immune deficiency syndrome, *STR* single tablet regimen

^a^Test available for 128 persons

^b^Other: 3 Personal choice of the participant, 1 expiration of the STP document (see main text) not intending to renew

**Fig. 3:**
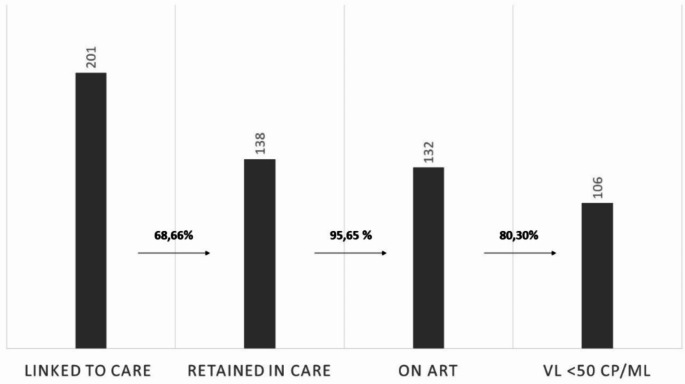
HIV clinical cascade according with 90-90-90 UNAIDS goals in migrant People Living With HIV taken in care from 01/01/2014 to 31/12/2022 (*N* = 201) at the Infectious and Tropical diseases unit in a tertiary teaching hospital in Florence, Italy (*ART* antiretroviral therapy, *VL* viral load, cp/mL)

**Fig. 4:**
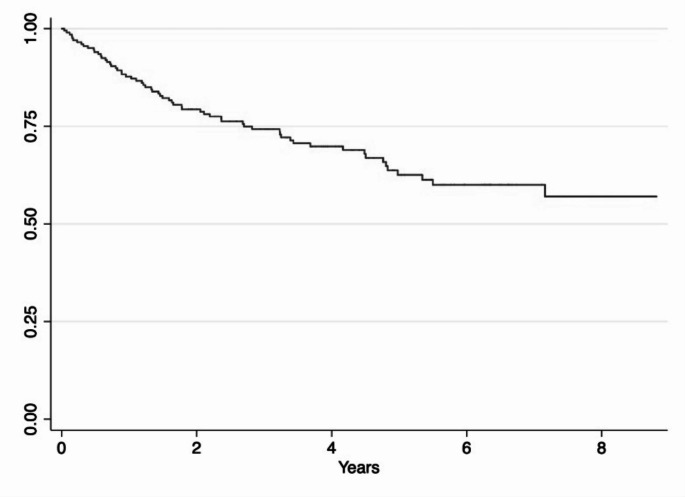
Kaplan Meier curve of incidence of loss to follow-up for all causes in migrant People Living With HIV taken in care from 01/01/2014 to 31/12/2022 (*N* = 201) at the Infectious and Tropical diseases unit in a tertiary teaching hospital in Florence, Italy

The study’s primary outcome was to assess the cascade of care, and we found that 68.7% (*n* = 138) of the enrolled migrants remained in care, with over 95.6% (*n* = 132) on ART and about 80% of them (*n* = 106) with undetectable viral loads (Fig. 3). The eight-year loss to follow-up incidence rate was 8.96 per 100 patients/year [CI95% 7.0–11.4], with further details in the Kaplan-Meier model shown in Fig. 4.

**Fig. 5:**
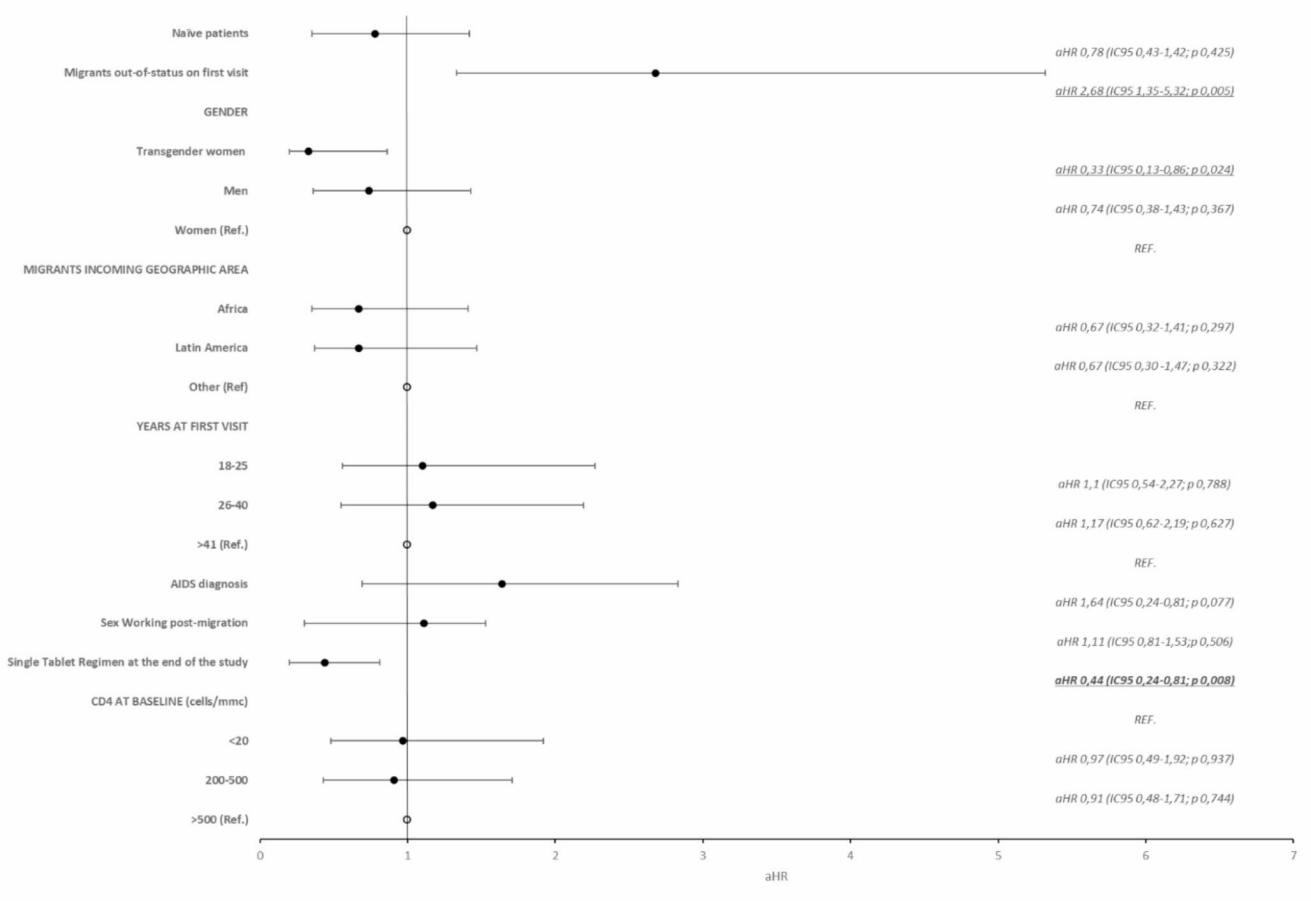
Forest plot of the multivariate analysis of predictors of loss at follow-up in migrant people living with HIV population taken in care from 01/01/2014 to 31/12/2022 (*N* = 201) at the Infectious and Tropical diseases unit in a tertiary teaching hospital in Florence, Italy

Multivariate analysis revealed that being out of status significantly increased the risk of loss to follow-up, with an adjusted hazard ratio (aHR) of 2.68 [1.35–5.32; *p* = 0.005]. Conversely, being a transgender woman and using a STR at the end of the study was associated with reduced risks of loss to follow-up, with aHRs of 0.33 [0.13–0.86; *p* = 0.024] and 0.44[0.24–0.81; *p* = 0.008], respectively (Fig. 5).

## Discussion

This study was conducted to better understand the health challenges and needs of migrants living with HIV (MLWH) referring to our center. With more than half of the patients enrolled coming from Latin America and a significant part from Peru, the data highlights Tuscany as the fourth region in Italy for the number of Peruvian migrants [21–22]. These findings diverge from other MLWH studies that focus predominantly on migrants from Sub-Saharan Africa, emphasizing the necessity of understanding local epidemiological trends [23–24]. Furthermore, approximately one-third of the study’s population comprised transgender women; the consistent representation of this population suggests the development of community-based care strategies as well documented in the literature [25–26].

In agreement with other studies [27–28–29], this study also found that the incidence rate of LTFU for HIV-positive migrants was high. Specifically, after 8 years, the LTFU incidence rate for migrants was 8.96 per 100 person-years, almost fourfold the rate for native Italians in the same setting previously reported [30–31].

More than half of the patients lost to follow-up were unreachable at the contact numbers provided, making it impossible to determine the reasons for their discontinuation. It is possible that fear of being sent back to their country of origin may discourage migrants from seeking medical attention, particularly if they face health complications. However, we believe this fear is mainly unfounded under the Italian universal healthcare system. The system does not impose any legal obligation to report out-of-status migrants. In cases of urgent medical needs, all individuals, regardless of legal status, receive the necessary care without the risk of being required to return to their home country. Although we do not have concrete data to confirm this, we think that the absence of such a reporting obligation is well-known among the migrant population living in Italy. This understanding should, in theory, help mitigate fears related to seeking healthcare services. Nonetheless, addressing and clarifying this misconception through targeted outreach and education might reduce the loss to follow-up cases among migrants. It is worth noting that we observed only one death during the extended follow-up period. This figure is undoubtedly underestimated, as we could not access data on the vital status of migrants lost to follow-up, particularly out-of-status.

Concerning the cascade of care, the UNAIDS 90-90-90 target has not yet been fully met at our center, with a proportion of patients “retained in care” of 68.7% and a proportion of MLWH virologically suppressed of 80%. Comparing this with the national average is currently tricky, as Italy and most other European nations cannot provide similar data on the migrant population. According to ECDC, in Europe in 2022, all four stages of the continuum of HIV care for migrants were reported only by four countries and at least two stages were reported only by nine countries [7]. However, based on these limited data, which are hard to compare with other European nations, our center reported viral suppression rates approximately 10% points lower (80 vs. 90%) probably because, post-arrival, migrants face cultural and language barriers, familial separation, and hurdles in employment and healthcare access, these obstacles could affect adherence to ART and follow-up visits with a consequent impact on viral suppression.

Besides, we tried to identify predictive factors for loss to follow-up within the study population. Being out-of-status emerged as a significant predictor of LTFU, echoing similar findings from studies in Milan [32].

To better understand this result we must consider that a migrant, in our country, without a regular residence permit does not have the right to enrol in the National Health Service but can access health services through the assignment of the STP code. Due to the linguistic and cultural barriers, this documentation is not always easy to obtain. It is not uncommon for pharmacological treatment to be suspended from the arrival of the migrant in Italy until they can request the STP. Additionally, the STP code is a code that holds validity for six months, and upon expiration, it must be renewed at designated offices during specific business hours. This underscores the necessity for tailored services to support this group’s legal, social, behavioural, and healthcare needs.

Conversely being a transgender woman and being on a STR regimen protects to LTFU. It is well known that a STR regimen positively influences retention in care and adherence; these regimens are indeed easier to take and generally preferred by patients, with lower rates of discontinuation compared to multi-pill regimens [33]. Nevertheless, being a transgender woman as a protective factor from LTFU seems to be in contrast with literature, but in Florence the presence of third-sector community, as street-unit volunteers and sex-workers care association, is effective and this is combined, in our center, with a healthcare trained staff in de-stigmatize language and clinical approach. Thanks to the collaboration of local associations, such as CAT (Cooperativa Sociale) and LILA (Lega Italiana per la Lotta contro l’AIDS), we were able to quickly identify a point of reference that provided legal assistance and information to all migrants seeking help within the National Health System [34]. A recent review underlines the role of the third sector [35] and concludes that a community-based approach is the key to improve the feel of belonging to a group and defines boundaries of stigma and isolation that an individual being a part of two key population, migrant and transgender, can experience. However, many transgender migrants in the study may be particularly motivated and adherent to healthcare protocols because they have migrated, at least in part, to access a better healthcare system. While their primary reason for migration may be to seek improved living conditions, the possibility of obtaining comprehensive medical support, including ART and hormone therapy, could also influence their commitment to care.

In their countries of origin, often in Latin America, structural barriers, limited resources, and social stigma hinder access to essential healthcare services. In contrast, Italy’s universal healthcare system offers these services more reliably, potentially encouraging stronger engagement with care among these individuals. Finally, we did not observe any differences when stratified by country of origin regarding the percentage of loss to follow-up. This could be due to the limitations present in our study. First, the small sample size, which, when analysing subpopulations, leads to excessive data granularity, preventing an accurate and sufficiently robust analysis. Moreover, it could also be because all migrant populations may face similarly difficult challenges and barriers to care retention, regardless of their country of origin. Moreover, due to its retrospective nature, unmeasured confounding factors have likely influenced the continuum of care process. These may include political, sociocultural, and linguistic barriers and the inherent nature of migrant status, which is characterized by a high propensity for movement and mobility. Lastly, since these data pertain to a single center and given that information on the migrant population is highly variable, with significant differences even between different centers within the same region, these data cannot be easily generalized to a broader context.

## Conclusions

In conclusion, we confirm a high prevalence of Peruvian migrants in Florence and a well-represented group of transgender women. UNAIDS goals have not yet been fully achieved in our setting, especially regarding retention in care. A high estimated incidence rate of loss to follow-up has been observed in migrants. Moreover, being an out-of-status migrant at the first visit is a predictor of loss to follow-up while being a transgender woman and using a STR Regimen at the end of the study are protective factors for loss to follow-up.

Although the data may not be completely generalizable to other contexts, the study still provides significant insights with an eight-year observation period. Further research should be performed in this field for reaching the ambitious goal of an efficient continuum of care especially in multifaced and vulnerable populations of migrants living with HIV that are often excluded from mainstream HIV treatment programs and experience specific barriers to healthcare.

## References

[1] International Organization For Migration (IOM). Migration and migrants: A global overview. In: Mcauliffe M, Triandafyllidou A, editors. World migration report 2022. Geneva: IOM; 2021.

[2] Mcauliffe M, Freier LF, Skeldon R, And Blower J. The great disrupter: Covid-19’s impact on migration, mobility and migrants globally. In: Mcauliffe M, Triandafyllidou A, editor. World migration report 2022. Geneva: International Organization For Migration (IOM); 2021.

[3] Carling J. ‘How Does Migration Arise?’. In McAuliffe M, Klein Solomon M (Conveners), Editors. Ideas to inform international cooperation on safe, orderly and regular migration. Geneva: IOM; 2017.

[4] Castro VA, King WM, Augustaitis L, Saylor K, Gamarel KE. A scoping review of health outcomes among transgender migrants. Transgend Health. 2022;7(5):385–96. 10.1089/trgh.2021.0011. 36644484 10.1089/trgh.2021.0011 PMC9829141

[5] Segala FV, Novara R, Panico G, Laforgia R, Raho L, Schiavone M, Civile G, Laforgia N, Di Gregorio S, Guido G, Cormio M, Dargenio A, Papagni R, L’Erario A, L’Erario L, Totaro V, Spada V, Valentini L, Frallonardo L, Lattanzio R, Falanga C, Putoto G, Saracino A, Di Gennaro F. Prevalence of sexually transmitted infections and predictors for loss to follow up among marginalized homeless and migrant communities: a cross-sectional study. Ann Glob Health. 2024;90(1):25. 10.5334/aogh.4388. 38618272 10.5334/aogh.4388 PMC11012089

[6] Santoso D, Asfia SKBM, Mello MB, Baggaley RC, Johnson CC, Chow EPF, Fairley CK, Ong JJ. HIV prevalence ratio of international migrants compared to their native-born counterparts: a systematic review and meta-analysis. EClinicalMedicine. 2022;53:101661. 10.1016/j.eclinm.2022.101661. 36147629 10.1016/j.eclinm.2022.101661 PMC9486043

[7] European Centre for Disease Prevention and Control. HIV and migrants. Monitoring implementation of the Dublin declaration on partnership to fight HIV/AIDS in Europe and central Asia: 2022 progress report. Stockholm: ECDC; 2023.

[8] Saracino A, Tartaglia A, Trillo G, Muschitiello C, Bellacosa C, Brindicci G, Monno L, Angarano G. Late presentation and loss to follow-up of immigrants newly diagnosed with HIV in the HAART era. J Immigr Minor Health. 2014;16(4):751–5. 10.1007/s10903-013-9863-z. 23943015 10.1007/s10903-013-9863-z

[9] Fakoya I, Álvarez-del Arco D, Woode-Owusu M, et al. A systematic review of post-migration acquisition of HIV among migrants from countries with generalised HIV epidemics living in Europe: implications for effectively managing HIV prevention programmes and policy. BMC Public Health. 2015;15:561. 10.1186/s12889-015-1852-9. 26085030 10.1186/s12889-015-1852-9 PMC4472169

[10] Drew RS, Rice B, Rüütel K, Delpech V, Attawell KA, Hales DK, Velasco C, Amato-Gauci AJ, Pharris A, Tavoschi L, Noori T. HIV continuum of care in Europe and central Asia. HIV Med. 2017;18(7):490–9. 10.1111/hiv.12480. 28117527 10.1111/hiv.12480 PMC5516161

[11] Sidibé M, Loures L, Samb B. The UNAIDS 90-90-90 target: a clear choice for ending AIDS and for sustainable health and development. J Int AIDS Soc. 2016;19:21133. 10.7448/IAS.19.1.21133. 27424601 10.7448/IAS.19.1.21133 PMC4947868

[12] UNAIDS Joint United Nations Programme on HIV/AIDS; Prevailing Against Pandemics By Putting People. At The Centre, Nov 2020, Geneve; prevailing-against-pandemics_en.pdf (unaids.org).

[13] European Centre for Disease Prevention and Control/WHO Regional Office for Europe. HIV/AIDS surveillance in Europe 2023–2022 data. Stockholm: ECDC; 2023.

[14] Dray-Spira R, Spire B, Heard I, Lert F, VESPA Study Group. Heterogeneous response to HAART across a diverse population of people living with HIV: results from the ANRS-EN12-VESPA study. AIDS. 2007;21(Suppl 1):S5–12. 10.1097/01.aids.0000255079.39352.9b. 17159588 10.1097/01.aids.0000255079.39352.9b

[15] Saracino A, Lorenzini P, Lo Caputo S, Girardi E, Castelli F, Bonfanti P, Galli M, Caramello P, Abrescia N, Mussini C, Monno L, Monforte AD. Increased risk of virological failure to the first antiretroviral regimen in HIV-infected migrants compared to natives: data from the ICONA cohort. J Int AIDS Soc. 2014;17(4 Suppl 3):19769. 10.7448/IAS.17.4.19769. 25397513 10.7448/IAS.17.4.19769 PMC4225436

[16] Saracino A, Lorenzini P, Lo Caputo S, Girardi E, Castelli F, Bonfanti P, Rusconi S, Caramello P, Abrescia N, Mussini C, Monno L, d’Arminio Monforte A, ICONA Foundation Study Group. Increased risk of virologic failure to the first antiretroviral regimen in HIV-infected migrants compared to Natives: data from the ICONA cohort. Clin Microbiol Infect. 2016;22(3):e2881–8. 10.1016/j.cmi.2015.10.026. 10.1016/j.cmi.2015.10.026 26551839

[17] Gagliardini R, Giacomelli A, Bozzi G, D’Arminio Monforte A, Tavelli A, Mazzotta V, Bruzzesi E, Cervo A, Saracino A, Mussini C, Girardi E, Cozzi-Lepri A, Antinori A, ICONA Foundation study group. Impact of COVID-19 pandemic on retention in care of native and migrant people with HIV in the ICONA cohort. Travel Med Infect Dis. 2024;58:102691. 10.1016/j.tmaid.2024.102691. 38336335 10.1016/j.tmaid.2024.102691

[18] Zucman D, Rasnaama A, Majerholc C, Vallée A. The COVID-19 pandemic and the migrant population for HIV diagnosis and care follow-up: they are left behind. Healthc (Basel). 2022;10(9):1607. 10.3390/healthcare10091607. 10.3390/healthcare10091607 PMC9498780 36141219

[19] Arora A, Quesnel-Vallee A, Lessard D, Mate K, Rodriguez-Cruz A, Kronfli N, Engler K, Vedel I, Lebouché B. Antiviral speed access program (ASAP) migrant advisory committee. Barriers and facilitators associated with steps of the HIV care cascade for migrants in OECD countries: a systematic mixed studies review protocol. BMJ Open. 2020;10(11):e040646. 10.1136/bmjopen-2020-040646. 33158835 10.1136/bmjopen-2020-040646 PMC7651739

[20] Vasylyeva TI, Horyniak D, Bojorquez I, Pham MD. Left behind on the path to 90-90-90: understanding and responding to HIV among displaced people. J Int AIDS Soc. 2022;25(11):e26031. 10.1002/jia2.26031. 36352546 10.1002/jia2.26031 PMC9646984

[21] La presenza dei migranti nella Città Metropolitana di Firenze. Direzione Generale dell’immigrazione e delle politiche di integrazione del Ministero del Lavoro e delle Politiche Sociali, Ministero del Lavoro e delle Politiche Sociali, Agenzia Nazionale delle Politiche Attive del Lavoro Servizi S.p.A. (ANPAL), 2022.

[22] Ministero del Lavoro e delle Politiche Sociali. La comunità Peruviana in Italia. Rapporto annuale Sulla presenza dei migranti. Area Servizi Per l’Integrazione ANPAL Servizi, 2022; https://www.integrazionemigranti.gov.it/AnteprimaPDF.aspx?id=5850. Accessed 02 Feb 2024.

[23] Yin Z, Brown AE, Rice BD, Marrone G, Sönnerborg A, Suligoi B, Sasse A, Van Beckhoven D, Noori T, Regine V, Delpech VC. Post-migration acquisition of HIV: estimates from four European countries, 2007 to 2016. Euro Surveill. 2021;26(33):2000161. 10.2807/1560-7917.ES.2021.26.33.2000161. 34414881 10.2807/1560-7917.ES.2021.26.33.2000161 PMC8380976

[24] Pezzoli MC, Hamad IE, Scarcella C, Vassallo F, Speziani F, Cristini G, Scolari C, Suligoi B, Luzi AM, Bernasconi D, Lichtner M, Cassara G, Manca N, Carosi G, Castelli F, PRISHMA Study Group. HIV infection among illegal migrants, Italy, 2004–2007. Emerg Infect Dis. 2009;15(11):1802–4. 10.3201/eid1511.090908. 19891869 10.3201/eid1511.090908 PMC2857252

[25] Donisi A, Colpani A, Zauli B, De Vito A, Fiore V, Babudieri S, Madeddu G. Sexually transmitted infections prevalence and cascade of care among undocumented sex workers: a twenty-year-long experience. Life (Basel). 2023;13(3):606. 10.3390/life13030606. 36983762 10.3390/life13030606 PMC10056054

[26] Navaza B, Abarca B, Bisoffi F, Pool R, Roura M, Provider-Initiated HIV. Testing for migrants in Spain: a qualitative study with health care workers and foreign-born sexual minorities. PLoS ONE. 2016;11(2):e0150223. 10.1371/journal.pone.0150223. 26914023 10.1371/journal.pone.0150223 PMC4767226

[27] Marukutira T, Gray RT, Douglass C, El-Hayek C, Moreira C, Asselin J, Donovan B, Vickers T, Spelman T, Crowe S, Guy R, Stoove M, Hellard M. Gaps in the HIV diagnosis and care cascade for migrants in Australia, 2013–2017: a cross-sectional study. PLoS Med. 2020;17(3):e1003044. 10.1371/journal.pmed.1003044. 32155145 10.1371/journal.pmed.1003044 PMC7064172

[28] Levison JH, Bogart LM, Khan IF, Mejia D, Amaro H, Alegría M, Safren S. Where it falls apart: barriers to retention in HIV care in Latino immigrants and migrants. AIDS Patient Care STDS. 2017;31(9):394–405. 10.1089/apc.2017.0084. 28891715 10.1089/apc.2017.0084 PMC5610398

[29] Ross J, Cunningham CO, Hanna DB. HIV outcomes among migrants from low-income and middle-income countries living in high-income countries: a review of recent evidence. Curr Opin Infect Dis. 2018;31(1):25–32. 10.1097/QCO.0000000000000415. 29095720 10.1097/QCO.0000000000000415 PMC5750122

[30] Lagi F, Kiros ST, Di Giambenedetto S, Lombardi F, Pecorari M, Borghi V, Lepore L, Monno L, Setti M, Micheli V, Bagnarelli P, Paolini E, Bai F, Bartoloni A, Sterrantino G. Long-term maintenance of virologic suppression in native and migrant HIV-1 Naïve patients: an Italian cohort study. AIDS Care. 2021;33(9):1159–66. 10.1080/09540121.2020.1839011. 33172289 10.1080/09540121.2020.1839011

[31] Lagi F, Kiros ST, Campolmi I, Giachè S, Rogasi PG, Mazzetti M, Bartalesi F, Trotta M, Nizzoli P, Bartoloni A, Sterrantino G. Continuum of care among HIV-1 positive patients in a single center in Italy (2007–2017). Patient Prefer Adher. 2018;12:2545–51. 10.2147/PPA.S180736. 10.2147/PPA.S180736 PMC6280894 30555224

[32] Ridolfo AL, Oreni L, Vassalini P, Resnati C, Bozzi G, Milazzo L, Antinori S, Rusconi S, Galli M. Effect of legal status on the early treatment outcomes of migrants beginning combined antiretroviral therapy at an outpatient clinic in Milan, Italy. J Acquir Immune Defic Syndr. 2017;75(3):315–21. 10.1097/QAI.0000000000001388. 28418991 10.1097/QAI.0000000000001388

[33] Clay PG, Yuet WC, Moecklinghoff CH, Duchesne I, Tronczyński KL, Shah S, Shao D. A meta-analysis comparing 48-week treatment outcomes of single and multi-tablet antiretroviral regimens for the treatment of people living with HIV. AIDS Res Ther. 2018;15(1):17. 10.1186/s12981-018-0204-0. 30373620 10.1186/s12981-018-0204-0 PMC6206661

[34] Lagi F, Gatteschi C, Tilli M, Zocco N, Avarello A, Bellini S, Ierardi F. Facilitators and barriers in HIV testing and continuum of care among migrant transgender women who are sex workers residing in Florence, Italy. Int J Transgend Health. 2023. 10.1080/26895269.2023.2209072. 10.1080/26895269.2023.2209072 PMC11044723 38681492

[35] Goldhammer H, Marc LG, Psihopaidas D, Chavis NS, Massaquoi M, Cahill S, Rebchook G, Reisner S, Mayer KA, Cohen SM, Keuroghlian AS. HIV care continuum interventions for transgender women: a topical review. Public Health Rep. 2023;138(1):19–30. 10.1177/00333549211065517 10.1177/00333549211065517 PMC9730173 35060802

